# The effect of anxiety and its interplay with social cues when perceiving aggressive behaviours

**DOI:** 10.1177/17470218241258209

**Published:** 2024-07-26

**Authors:** Fábio Silva, Marta I. Garrido, Sandra C. Soares

**Affiliations:** 1 William James Center for Research, University of Aveiro, Aveiro, Portugal; 2 Melbourne School of Psychological Sciences, The University of Melbourne, Vic, Australia; 3 Graeme Clark Institute for Biomedical Engineering, The University of Melbourne, Vic, Australia

**Keywords:** Anxiety, threat, visual perception, social perception, expectations

## Abstract

Contextual cues and emotional states carry expectations and biases that are used to attribute meaning to what we see. In addition, emotional states, such as anxiety, shape our visual systems, increasing overall, and particularly threat-related, sensitivity. It remains unclear, however, how anxiety interacts with additional cues when categorising sensory input. This is especially important in social scenarios where ambiguous gestures are commonplace, thus requiring the integration of cues for a proper interpretation. To this end, we decided to assess how states of anxiety might bias the perception of potentially aggressive social interactions, and how external cues are incorporated in this process. Participants (*N* = 71) were tasked with signalling the presence of aggression in ambiguous social interactions. Simultaneously, an observer (facial expression) reacted (by showing an emotional expression) to this interaction. Importantly, participants performed this task under safety and threat of shock conditions. Decision measures and eye-tracking data were collected. Our results showed that threat of shock did not affect sensitivity nor criterion when detecting aggressive interactions. The same pattern was observed for response times. Drift diffusion modelling analysis, however, suggested quicker evidence accumulation when under threat. Finally, dwell times over the observer were higher when under threat, indicating a possible association between anxiety states and a bias towards potentially threat-related indicators. Future probing into this topic remains a necessity to better explain the current findings.

## Introduction

The way we see the world is subject to a constant, but ever-changing, filter of influences. Some of these influences can be more accurately identified, such as the influence of alcohol on perception (Calhoun et al., 2004), while others are less obvious. These latter ones are the result of internal cognitive processes that integrate external information, such as our context and prior experiences, and internal states themselves, such as motivations and emotional states. Taken together, these form the basis of the expectations that we use in attributing meaning (interpreting) to our visual world (de Lange et al., 2018; Dunning & Balcetis, 2013).

The benefits of expectations are mostly seen when the quality of our sensory input is ambiguous or degraded—that is, less reliable (de Lange et al., 2018). This happens when one is clearly aware of the limited (degraded) sensory input, such as when interpreting a conversation in a loud place, or under more subtle conditions that require us to resolve ambiguous interpretations. This is particularly the case during social interactions, since communication is riddled with subtle nuances that rapidly change the meaning/intent of the communicator (Campbell et al., 2022; Friston & Frith, 2015). In these cases, context provides an important aid when interpreting our social environment (Manera et al., 2011; Zillekens et al., 2018).

Likewise, emotional states can also alter our visual system and bias expectations, shaping how we view our world (Gasper & Clore, 2002; Riener et al., 2011; Stefanucci et al., 2008), and in specific, our social environments (Jolij & Meurs, 2011; Niedenthal et al., 2000; Niedenthal & Wood, 2019). Here, we focus on the specific affective state of anxiety and how it affects visual perception. These changes have already been documented in relation to high-trait anxiety (e.g., Gray et al., 2009; Rossignol et al., 2005), but remain less explored in the case of functional anxiety states. A few studies have shown that states of anxiety enhance sensory–perceptual processing, leading to a rougher, but quicker (even if, perhaps, more error-prone), detection of salient stimuli (Lojowska et al., 2015, 2019; Robinson, Vytal, et al., 2013). For example, when faced with repeating patterns of sensory input, deviants generate greater mismatch negativity (MMN) event-related potentials when under threat (Cornwell et al.,2007, 2017; but see Fucci et al., 2019). This gain is even shown to take place prior to any high-order (cortical) processing of the sensory information (Baas et al., 2006).

An additional characteristic of this increased sensory–perceptual processing seen during anxiety is the fact that it is predominantly directed at threat-signalling stimuli (Robinson et al., 2011; Sussman et al., 2016). For instance, in line with the increased MMN research exemplified above, there is an increased response to fearful (but not happy) facial expressions in the ventral striatum when such faces are unexpected (Robinson, Overstreet, et al., 2013). Other studies have come to support the idea that, just as in high-trait or pathological anxiety (Bui et al., 2017; Capitão et al., 2014), one’s sensitivity towards a threat-related stimulus is increased when under threat, leading to quicker and/or more accurate detections (Sussman et al., 2016; see Bar-Haim et al., 2007). Aside from being identified quicker, facial expressions that signal threat (i.e., fear and anger) are also perceived as being more intense and more easily judged as fearful (Flechsenhar et al., 2022; Kavcıoğlu et al., 2019; Wudarczyk et al., 2016; Zhou & Chen, 2009).

One question that has gotten little attention, however, concerns how we incorporate contextual cues, namely those that can be used to signal threat or lack thereof, under states of anxiety. This is of particular importance since an enhanced sensory-driven perception, typical of such states, consequently, implies a lesser dependency on expectations (prior information). In turn, this is supposed to result in a reduced specificity when interpreting and discriminating visual information (Cornwell et al., 2007, 2017; van Marle et al., 2009). However, an earlier study conducted by the authors (Silva et al., 2024) has failed to evidence shifts in participants’ perception under anxiety when judging ambiguous social interactions. Crucially, all the visual elements in this task, both contextual cues and target stimulus, were emotionally neutral. A natural follow-up question, given the association between threat-related sensitivity and anxiety, concerns how emotional social scenes are perceived under threat. Namely, how are both sensitivity and criterion (related to specificity) measures affected by anxious states when the social stimuli have a threat-related emotional appraisal.

The idea that under anxiety, the presence of other threat-related factors (e.g., a fearful face as opposed to a neutral one) further potentiates anxiety-related effects has already been suggested (Grillon & Charney, 2011; see also Wieser & Keil, 2014). Sussman and colleagues (2016) explored this by measuring if when under anxiety, compared with safe conditions, participants benefitted from being exposed to a fearful cue when identifying fearful expressions (among neutral expressions). They showed a greater perceptual sensitivity under threat in detecting fearful expressions compared with safe conditions, with this effect being conditional on high levels of trait anxiety. However, by not comparing different types of priming cues (fear vs. neutral) when detecting fearful expressions, no conclusions concerning how the identification of threat is boosted by fearful cues seems yet warranted. Furthermore, no measurement of criterion was calculated, thus not revealing the extent of any general bias (expected likelihood of fearful expression) induced by the context and cues.

Thus, in this study, our aim is twofold: (1) explore the decision parameters (sensitivity and criterion) related to the identification of aggression when under an anxiety state and (2) assess how emotional cues are incorporated in the perception of aggression in ambiguous social scenes when under anxiety. To this end, we used ambiguous social displays that could either convey an aggressive (anger gesturing) or an innocuous interaction. In addition, we paired these scenes with an external agent (observer) that was depicted as reacting to the interaction taking place. By manipulating its facial expression, either showing a fearful reaction or a neutral facial expression, we created contextual cues that participants were exposed to before judging the nature of the scene taking place (aggressive or not). Importantly, participants undertook this task while under safe and threat of shock conditions. We collected response data to establish their sensitivity (d′) and criterion (c), as well as response times (RTs) and gaze data.

With this in mind, and considering the literature discussed above showing that anxiety improves perceptual sensitivity towards threat, we expected the following: when under threat (compared with the safe condition) participants would exhibit (1) an overall higher sensitivity in detecting aggression; (2) a bigger gain in sensitivity when presented/primed with a fearful facial expression (compared with a neutral one); (3) an overall reduced criterion (general tendency to report aggression); and finally (4) quicker reaction times. The remaining comparisons involving anxiety inventories as well as gaze data, will be interpreted as exploratory analyses.

## Method

### Participants

Sample size was determined with the use of Superpower package for R (v. 0.2.0; Lakens & Caldwell, 2021). Based on the prior literature, we hypothesised expected means and a standard deviation for our analysis design (2 × 2 within-subjects), running a total of 2,000 simulations. We arrived at a minimum of 62 participants for a desired power of .8 (and a partial eta-squared of .15). To account for possible exclusions, we collected a total of 73 participants, mostly university students. Participants were only included if they had no current psychiatric/neurological disorders and were not currently under any medication related to anxiety/depression or with clear implications over mood/cognitive functions. Of these, one was removed for having more than 25% of the trials with no-response. Another was removed for showing a threat-identification accuracy in the final recognition task below 75%. No highly abnormal patterns regarding facial expression identification were found (no participants removed). Our final sample consisted of 71 participants (57 females; *M*_age_ = 21.5, *SD*_age_ = 3.9). The present study was conducted with permission from the ethics committee (reference 02-CED/2021) and in accordance with the Declaration of Helsinki and data protection regulation from the University of Aveiro.

### Stimuli and apparatus

The point-light displays of agents performing different actions were gathered and generated with the Social Perception and Interaction Database (Okruszek & Chrustowicz, 2020). We select “Altercation,” “Denying accusations,” “Stopping the conversation,” and “Taking the blame” for our aggressive set of actions and “Come close,” “Give me that,” “Look there,” and “Pick it up” for our neutral set (*M*_duration_ = 3.8 s, *SD*_duration_ = 0.4 s). The actions “Confronting an aggressor” (aggressive) and “Sit down” (neutral) were used in the practice phase. Each action (used in main task) was generated with a flicker (limited lifetime technique) set at six points, with asynchronous appearance and disappearance varying between 150 and 250 ms. To avoid familiarity due to repeated exposure, each action (and accompanying flickering pattern) with six different versions per action were generated. Each video was presented in a window of 1,280 by 720 pixels. Considering the chin-rest position (60 cm away from the screen), the effective size of the area occupied by the agents was 19 × 11.4 visual degrees.

**Figure 1.**
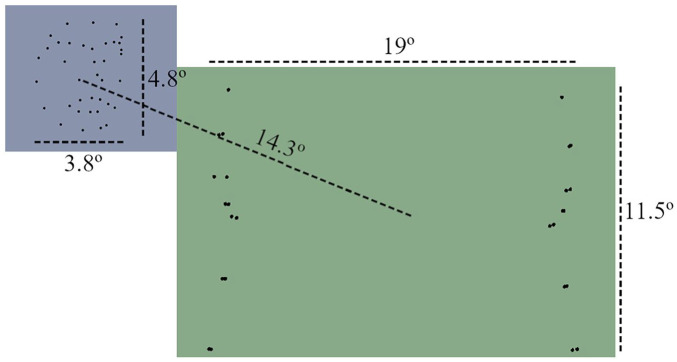
Scheme of the overall disposition of the stimuli, their respective occupied area (in visual degrees), and their regions of interest (ROIs). The blue colour represents the face ROI and the green colour represents the central/main action ROI.

The fearful and neutral facial expressions were retrieved from the PLAViMoP database (Decatoire et al., 2019). Each expression (fearful and neutral) was manipulated so as to present a ~30º angle towards the left and the right. This angle allowed the faces to be perceived as directed towards the interaction that would take place in the middle of the screen (see Figure 1). Each video was presented in a window of 512 × 288 pixels. The faces occupied an area of 3.8 × 4.8 visual degrees and were positioned at around 14.3 visual degrees away (diagonally) from the middle of the screen (see Figure 1).

The electric shocks, used to induce anticipatory anxiety, were delivered with the STMISOLA module from Biopac. The shocks ranged from 2 to 6 mA and had a duration of 100 ms. The two electrodes were attached to the participants’ forearm, with a distance of about 3 cm between them.

The experimental task was displayed on an MSI Pro MP241 monitor with a 1,920 × 1,080 pixel resolution. Behavioural responses were given through a standard QWERTY keyboard. The data from online questionnaires were collected using Limesurvey (forms.ua) and the experimental task was programmed using Psychopy version 2021.2.3 (Peirce et al., 2019). The eye tracker used was a Gazepoint GP3 (150 Hz).

### Procedure

Recruitment took place via a brief online form, where participants, after providing their informed consent, filled out socio-demographic information (age, sex, etc.), and completed the trait portion of the State-Trait Inventory for Cognitive and Somatic Anxiety (STICSA; Barros et al., 2022; Ree et al., 2008) and the Liebowitz Social Anxiety Scale (LSAS; Caballo et al., 2019; Liebowitz, 1987).

The experimental session, conducted in a lab, began with an initial description of the study and the informed consent. Participants were then asked to fill in the state portion of the STICSA. The shock workup procedure followed, with two electrodes being applied on the left forearm of the participant. Here, they received a graded series of electric shocks, starting at 2 mA and going up to a maximum of 6 mA. Each shock was followed by a question (to be answered on a Likert-type scale) concerning how unpleasant the shock they had just received had felt on a scale from 1 (*barely*) to 5 (*very unpleasant/uncomfortable*). The shock intensity was increased in steps of 1 mA until the rating of 4 (*quite unpleasant/uncomfortable*) or the maximum intensity level was reached. The calibrated intensity for each participant was used throughout the rest of the experiment. Importantly, if the rating of 4 was reached before the 6-mA maximum level, electric shocks of that same intensity were administered until a total of five shocks (since the beginning of the workup procedure) were delivered.

Participants were positioned in front of the lab computer, and, with their head supported on a chinrest, performed a brief eye-tracker calibration. Two more calibrations would occur, one at the start and another at the middle point of the main task (after, roughly, 10 min).

A practice phase followed. Each trial began with the presentation of an external observer (face) followed by a central video of two agents interacting with each other. Participants were asked to initially focus their attention on the external observer, who could appear at the upper left or upper right quadrants of the screen (indicated by a prior loading/fixation cross). They were instructed to only redirect their attention to the central interaction, after being shown the two central agents (1.3 s after the trial began). The observer would only be “looking” towards the central interaction, but not showing any type of emotional expression. The participants’ task was to identify (keypress) if the central action between the two agents was aggressive or not, as quickly and accurately as possible. Their response was to be given during the video or up to 1 s (blank screen) after the video ended. The observer remained on-screen until the end of the video. As previously mentioned, the actions presented during this practice phase were only displayed with a reduced amount of flickering to facilitate identification. Importantly, the task was completed in two different types of blocks (two trials per block: one safe and one aggressive action). In one block (safe), participants were told that they would not be receiving any electric shock. In the other block (threat), they were informed that they would receive one electric shock at any moment. These two blocks were accompanied by two lateral rectangles that would either be empty (only white outline; safe block) or coloured in yellow (threat block). In addition, participants received feedback (regarding their accuracy) after each practice trial (this was not the case in the main task).

**Figure 2.**
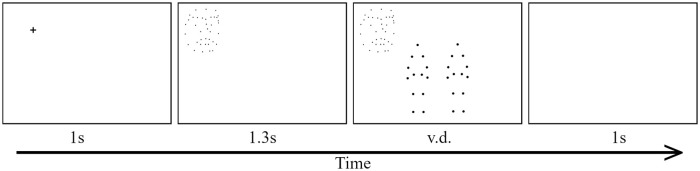
General illustration of a trial. Lateral rectangles are omitted. Participants had a 1-s fixation cross. This cross could be shown in the left (as in the picture) or right upper portions of the screen. This cross was replaced by a face (observer) which could either show a facial expression or remain neutral. After 1.3 s, the central video (with a 0.2-s fade-in) was presented. After the video ended, if no answer was provided, they were shown a blank screen for 1 s, giving the participants extra time to provide their answers. v.d.: video duration.

In the main task (see Figure 2 for an illustration of a trial), participants were asked to follow the same instructions as before. However, they were told that this time the observer would be reacting to the central action that would be presented after (and alongside) him. In addition, this time the actions would be more difficult to identify (full flickering). Blocks were also longer, and, in the threat block, participants were told that they could receive a random number of electric shocks. However, unbeknownst to the participants, they would only receive two shocks per block (six in total; 8% of the trials). Each block was composed of a total of 32 trials, with each action being represented a total of 4 times (accounting for different observer emotions and positions). The blocks alternated between safe and threat blocks, with the starting block being counterbalanced per participant. Importantly, to ensure that participants were paying attention to the observer at the start of each trial, in two random trials per block they were asked to identify if the observer had exhibited any facial expression. At the end of each block, they were also asked to indicate, in a visual analog scale (0–100), their experienced anxiety during that block. At the middle mark of the main task, they took a small break.

After the main task, participants had the brief additional tasks/questions. In the first one, they had to identify the emotion associated with each action observed during the main task (now without any flicker/limited-lifetime technique). The second task asked participants to rate what emotions best described each facial expression (neutral and fearful) shown by the observer (both left- and right-oriented versions) during the task. They did do so by, after watching each video of the observer, rating how each emotion (see the online Supplementary Material B) was associated with the video (facial expression of the observer) that they had just watched. Their responses to each emotion were given in a visual analog scale (0–100). Finally, they were asked if they felt that the relationship between the observer (facial expression) and the central action was different between blocks (threat and safe) and, if so, in which block they believed the association was higher. This marked the end of the experiment, and participants were then debriefed and thanked for participating.

### Statistical analysis

All data treatment and statistical analyses were performed with R (2022.02.1). Prior to any analysis, data from trials containing no response were removed (around 3% of all trials), as well as trials where RTs were inferior to 1 s after the central action appeared (around 1% of all trials). As measures of effect size, we used partial omega-squared (ω_p_^2^) for ANOVAs and Cohen’s *d* (*d*) for the *t-*tests. All measures, manipulations, and exclusions are reported here.

Signal detection theory measures of sensitivity and criterion were acquired with the psycho package (0.6.1; Makowski, 2018). A *p*-value below .05 was set for statistically significant effects. Normal distribution of residuals was assessed visually and with Shapiro–Wilk tests. All post hoc analysis used Bonferroni corrected *p*-values.

Sensitivity, criterion, and RT were analysed with repeated measure ANOVAs. The introduction of covariates was considered (STICSA-State, STICSA-Trait, and LSAS), but the best models, as measured by model *p*-values and Akaike information criterion (AIC) values, were those without any covariate. For RT analysis, only correct trials were considered. To additionally provide support for the null hypothesis in our main analyses, Bayesian analyses were performed (Supplementary Material A) with their respective Bayes factors (BF) being reported in the main results section.

We also performed a drift diffusion modelling (DDM) analysis to allow both RTs and decision criteria into one single analysis. Here, the RT variable was transformed to start only after the first fixation over the region of interest (ROI) containing the central action in each trial (and not since the beginning of the video). This allowed us a clearer measurement on initial attention over the target (central action). Given the track loss experienced in some of the trials, around 3% of trials were excluded from these analyses, since in these cases we were unable to derive the time of first fixation. In addition, around 2% of the trials were excluded due to first fixation times superior to 1 s after action onset, indicating either inattentiveness or calibration problems. The DDM parameter estimation was performed with the Fast-dm software (v. 30.2; Voss & Voss, 2007), using maximum likelihood as a computation method (precision at 4.0). Parameters for boundary separation (alpha) and starting point (z) were estimated separately per block and facial emotion, while drift rate was additionally estimated for the type of action (aggressive versus neutral). Drift rate was analysed as a function of magnitude towards the correct response (values were transformed accordingly; Myers et al., 2022). Given our prolonged trial responses, and limited number of trials, the non-decision time was fixed at 0.3 (default of the software) for every individual and condition, and all other parameters concerning intertrial variability of parameters were fixed at zero (Lerche & Voss, 2016).

Concerning eye-tracking data, trials with track loss (loss of correct tracking of the participants eye) superior to 25% were removed, resulting in the exclusion of 237 trials (1.7% of the data). Due to calibration issues, one participant was removed due to an overall track loss of over 25%. The final number of participants in the eye-tracking data was 70. Data transformation for window time and sequential analyses was performed with the package *eyetrackingR* (0.2.0; Dink & Ferguson, 2015) for R. For the proportion analysis, data were binned into 200-ms intervals and analysed in a generalised linear mixed model with a beta family. In addition, the time window for analysis was set between 800 and 1,800 ms (centred at 1,300 ms, when the main action was shown). This model had block, face emotion, and time (centred) as fixed factors. Participant IDs were incorporated as random intercepts (no random slopes were added due to convergency issues).

When analysing time until first fixation over the central ROI (see Figure 1), only trials in which the first fixation was less than 1 s after central video onset (1.3-s mark) were considered. For the fixation duration and count analysis over the observer ROI, only fixations with a minimum duration of 50 ms were used (Rayner, 2009). The period for this analysis was established from the beginning of the trial until the appearance of the central action (1.3 s), plus the time it took for the participant to remove its gaze from the face/observer ROI. No fixation that went over the 2-s time mark of the trial was considered for this analysis. Fixation duration and count were analysed with linear mixed models and generalised linear mixed models (Poisson family), respectively. In both models, block was added as a fixed factor and the random structure of the model comprised random intercepts per participant ID, with varying slopes per block.

Descriptive and graphical analyses over the last questions of this experiment are described in the Supplementary Material B. This study was preregistered (osf.io/98vdg).

## Results

### Manipulation check

Analysis over reported values of anxiety showed that our threat manipulation worked as intended, with threat blocks eliciting greater feelings of anxiety (*M* = 38.1, *SD* = 25.2) compared with safe ones, *M* = 14.1, *SD* = 15.8; *t*(70) = 11, *p* < .001, *d* = 1.29, 95% CI = [0.97, 1.60].

### Sensitivity and criterion

Our analysis showed that threat of shock had no statistically significant effect over sensitivity, *F*(1, 70) = 1.782, *p* = .186, ω_p_^2^ = 0, 95% CI = [0, 0.06]; BF_01_ = 5.05. The same was observed for the facial expression of the observer, *F*(1, 70) = 0.004, *p* = .948, ω_p_^2^ = 0, 95% CI = [0, 0]; BF_01_ = 4.72, as well as the interaction between threat of shock and the latter, *F*(1, 70) = 0.076, *p* = .783, ω_p_^2^ = 0, 95% CI = [0, 0]; BF_01_ = 3.86.

**Figure 3.**
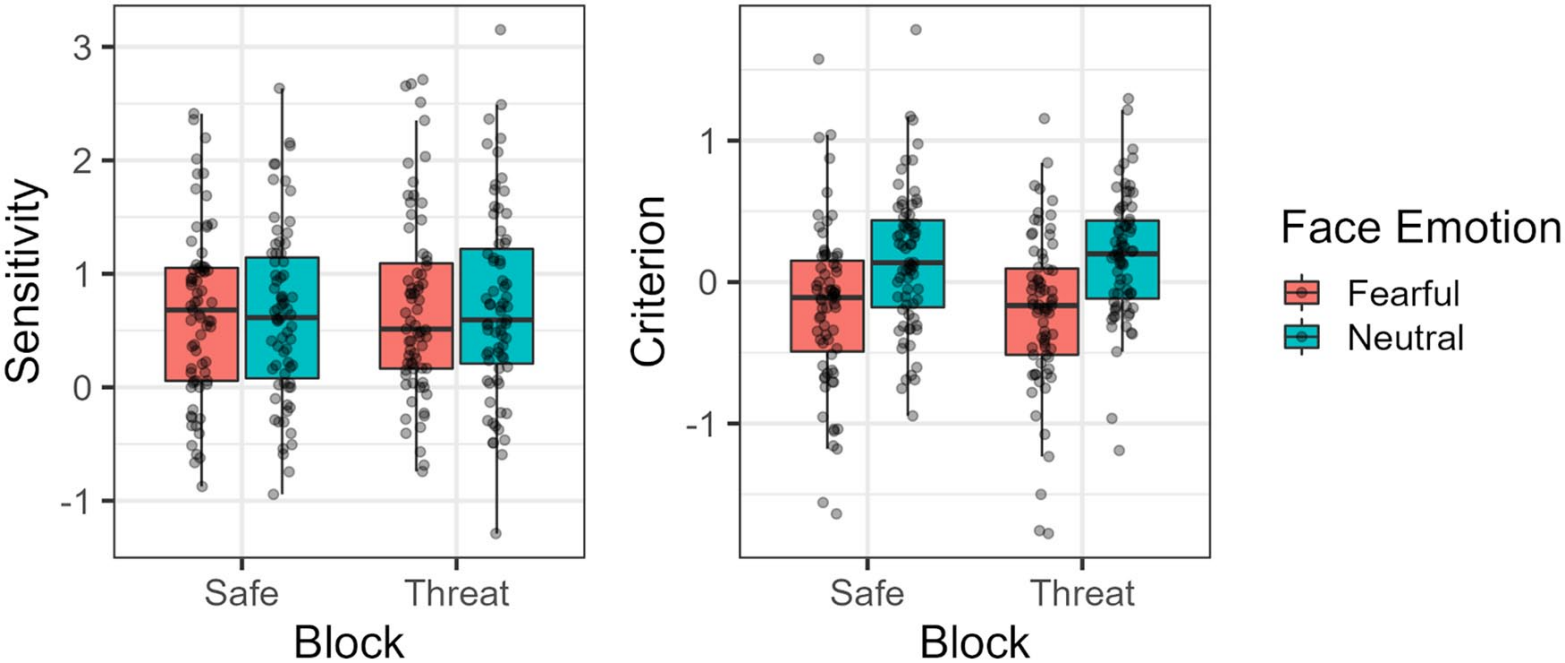
Observed sensitivity and criterion values per block and facial expression of the observer.

For criterion, threat also showed no statistically significant effect on this measure, *F*(1, 70) = 0.005, *p* = .943, ω_p_^2^ = 0, 95% CI = [0, 0]; BF_01_ = 6.71, while the facial expression of the observer did, *F*(1, 70) = 36.93, *p* < .001, ω_p_^2^ = 0.11, 95% CI = [0.01, 0.26]; BF_10_ = 73.14. In particular, fearful/surprise expressions led to lower criterion values compared with neutral expressions (see Figure 3). No interaction between threat of shock and facial expression of the observer was found, *F*(1, 70) = 1.87, *p* = .176, ω_p_^2^ = 0, 95% CI = [0, 0.05]; BF_01_ = 4.42.

In terms of correlations, sensitivity was shown not to have any statistically significant association with STICSA-Trait, *t*(68) = −0.07, *p* = .9, *r* = .009, 95% CI = [−0.226, 0.243]; STICSA-State, *t*(68) = −0.1, *p* = .9, *r* = −.016, 95% CI = [−0.25, 0.22]; and LSAS, *t*(68) = −.02, *p* > .99, *r* = −.002, 95% CI = [−0.237, 0.233]. The same conclusions are observed between criterion and STICSA-Trait, *t*(68) = −0.1, *p* = .9, *r* = −.013, 95% CI = [−0.247, 0.223]; STICSA-State, *t*(68) = 1, *p* = .2, *r* = .152, 95% CI = [−0.086, 0.373]; and LSAS, *t*(68) = −1, *p* = .3, *r* = −.118, 95% CI = [−0.344, 0.120].

### RTs

**Figure 4.**
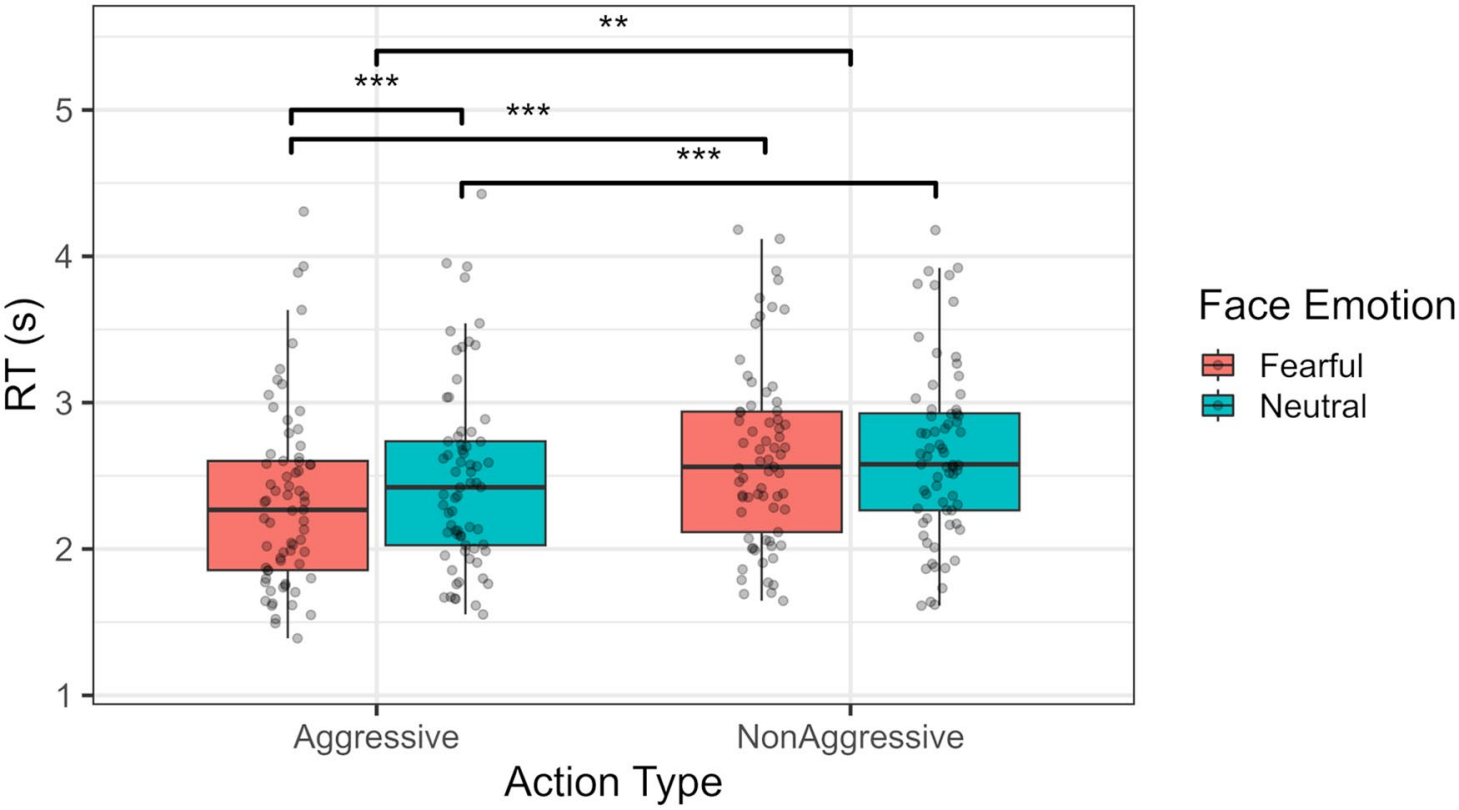
RT distribution per type of action (aggressive vs. non-aggressive) and face emotion (fearful vs. neutral). **p < .01. ***p < .001.

In terms of RTs, no apparent differences were found between threat and safe blocks, *F*(1, 68) = 0.067, *p* = .796, ω_p_^2^ = 0, 95% CI [0, 0]; BF_01_ = 7.19. However, RTs were, on average, faster for aggressive actions, compared with neutral ones, *F*(1, 68) = 39.717, *p* < .001, ω_p_^2^ = .034, 95% CI = [0, 0.15]; BF_10_ = 6, as well as for fearful compared with neutral expressions by the observer, *F*(1, 68) = 29.934, *p* < .001, ω_p_^2^ = .005, 95% CI = [0, 0.09]; BF_10_ = 2.9. No interaction between threat of shock and any of these variables was found (*p* *>* .05). However, face emotion did interact with type of action, *F*(1, 68) = 9.552, *p* = .003, ω_p_^2^ = .002, 95% CI = [0, 0.07]; BF_10_ = 4.76, with aggressive actions being detected quicker if preceded by fearful compared with neutral expressions, *t*(68) = −5.858, *p* < .001, *d* = −.234, 95% CI = [−0.36, −0.11]; see Figure 4. No significant three-way interaction was observed, *F*(1, 68) = 0.127, *p* = .723, ω_p_^2^ = 0, 95% CI = [0, 0]; BF_01_ = 3.05.

### DDM

**Figure 5.**
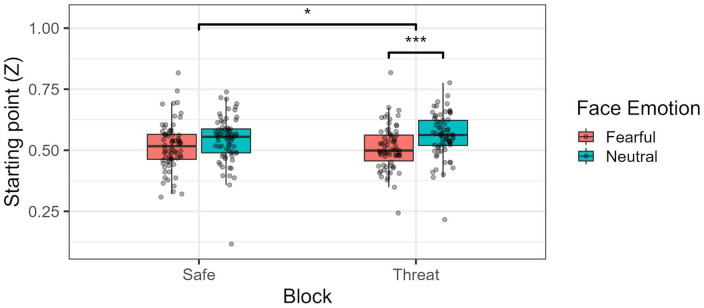
Generic DDM graphic, showing the estimated starting points (Z) and drift rates (D) per type of action, facial expression, and block across time. The *y* axis represents the boundary separation (α). M decision boundary was associated with a “non-aggressive” decision, while the “Z” boundary was associated with an “aggressive” decision. *p < .05. ***p < .001.

**Figure 6.**
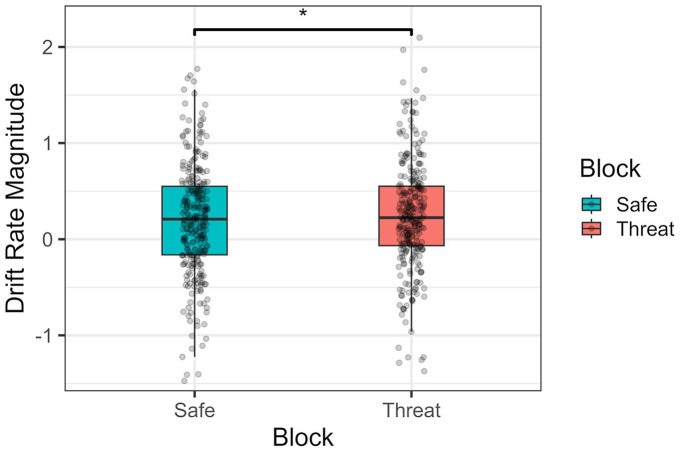
Starting point (Z) per block type and face emotion. Higher levels of starting point (above 0.5) mean a bias towards identifying the central action as aggressive. *p < .05. ***p < .001.

Regarding the DDM analysis (see Figure 5 for a general graphic view), in terms of boundary separation (α), no statistically significant difference was found between block types, *F*(1, 69) = 0.26, *p* = .61, ω_p_^2^ = 0, 95% CI = [0, 0]; face emotion, *F*(1, 69) = 3.34, *p* = .072, ω_p_^2^ = 0.001, 95% CI = [0, 0.06]; and their respective interaction, *F*(1, 69) = 1.18, *p* = .28, ω_p_^2^ = 0, 95% CI = [0, 0.01]. As for starting point, block alone did not significantly affect this measure, *F*(1, 69) = 0.88, *p* = .35, ω_p_^2^ = 0, 95% CI = [0, 0]. In the case of face emotion, however, we saw that, on average, participants had a higher starting point (towards signalling aggression) when the facial expression of the observer was neutral as opposed to fearful, *F*(1, 69) = 19.5, *p* < .001, ω_p_^2^ = 0.04, 95% CI = [0, 0.17]. In addition, the difference between these two facial expressions in terms of starting point was larger for threat blocks compared with safe blocks, *F*(1, 69) = 4.41, *p* = .039, ω_p_^2^ = 0.004, 95% CI = [0, 0.08]; see Figure 6. Post hoc comparisons showed that face emotion was significantly different in the threat blocks, *t*(123) = −4.830, *p* < .001, *d* = −.44, 95% CI = [−0.62, −0.25], but not in the safe blocks, *t*(123) = −2.430, *p* = .099, *d* = −.22, 95% CI = [−0.40, −0.04]. No difference between fearful emotion, *t*(137) = 0.74, *p* > .99, *d* = .06, 95% CI = [−0.1, 0.23], and neutral emotion, *t*(137) = −2.11, *p* = .218, *d* = −.18, 95% CI = [−0.35, −0.01] was found across blocks.

**Figure 7.**
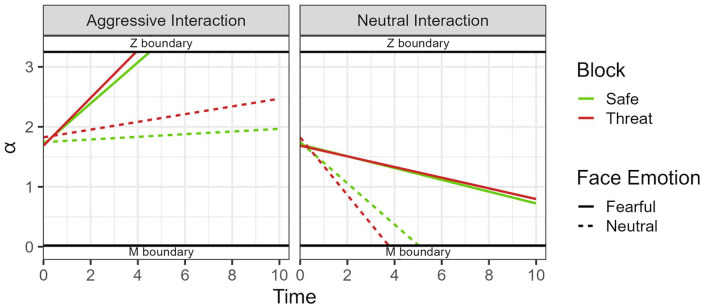
Absolute magnitude of the drift rate values per block type (threat-of-shock vs. neutral). Higher values indicate a quicker accumulation of evidence towards a final correct response. *p < .05.

In terms of drift rate magnitudes, threat of shock did significantly lead to bigger drift rates (faster evidence accumulation towards the correct response) compared with safe contexts, *F*(1, 69) = 4.72, *p* = .033, ω_p_^2^ = 0.007, 95% CI = [0, 0.09]; see Figure 7. No main effect of face emotion, *F*(1, 69) = 0.03, *p* = .85, ω_p_^2^ = 0, 95% CI = [0, 0], nor any effect of action type was observed, *F*(1, 69) = 0.25, *p* = .62, ω_p_^2^ = 0, 95% CI = [0, 0]. The effect of block was not statistically different across face emotion, *F*(1, 69) = 1.75, *p* = .19, ω_p_^2^ = 0.001, 95% CI = [0, 0.06], or action type, *F*(1, 69) = 0.07, *p* = .79, ω_p_^2^ = 0, 95% CI = [0, 0]. A significant interaction between face emotion and action type was present, *F*(1, 69) = 38.8, *p* < .001, ω_p_^2^ = 0.17, 95% CI = [0.04, 0.33], with bigger drift rates in aggressive actions when presented with a fearful expression, *t*(94.8) = 5.77, *p* < .001, *d* = .59, 95% CI = [0.37, 0.81], and in neutral actions when presented with a neutral expression, *t*(94.8) = −5.62, *p* < .001, *d* = −.58, 95% CI = [−0.79, −0.36]. No three-way interaction was observed, *F*(1, 69) = 2.67, *p* = .11, ω_p_^2^ = 0.003, 95% CI = [0, 0.08]).

### Eye tracker

**Figure 8.**
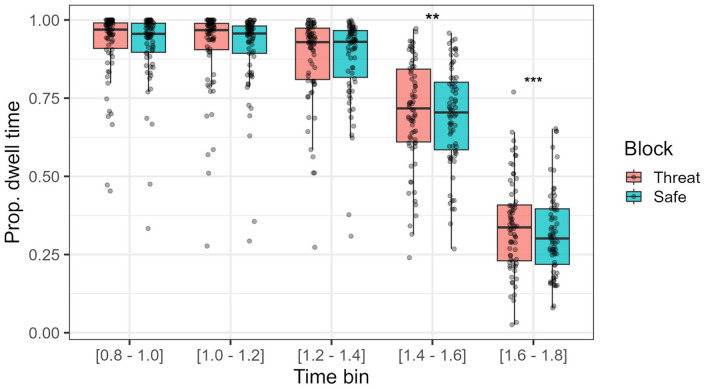
Proportion of dwell time spent on face emotion ROI compared with central ROI at each time bin (200-ms time bins). Higher dwell times signify more time spent gazing at the face ROI compared with the central ROI across trials and participants. **p < .01. ***p < .001.

While under threat (compared with safe conditions), participants showed a greater dwell time over the face ROI compared with the main/central action ROI between the 800 and 1,800 ms time mark (χ²(1) = 5.73, *p* = .017). In addition, fearful faces were associated with lower dwell time proportions (face ROI over central action ROI), compared with neutral faces (χ²(1) = 581.97, *p* < .001). As expected, the time spent gazing at the face ROI diminished across the 1-s time window (χ²(1) = 10,980, *p* < .001). No interaction was found between block and face emotion (χ²(1) = 1.27, *p* = .260), but the effect of block on dwell time proportion was significantly modulated by time bin (χ²(1) = 5.52, *p* = .019). Namely, as seen in Figure 8, participants under threat dedicated more time to the face ROI compared with the central ROI after the 1.3-s mark. The time variable also showed an interaction with face emotion (χ²(1) = 885.70, *p* < .001), with neutral faces having greater dwell times compared with fearful faces after the 1.3-s mark. No statistically significant three-way interaction was found (χ²(1) = 0.14, *p* = .705).

**Figure 9.**
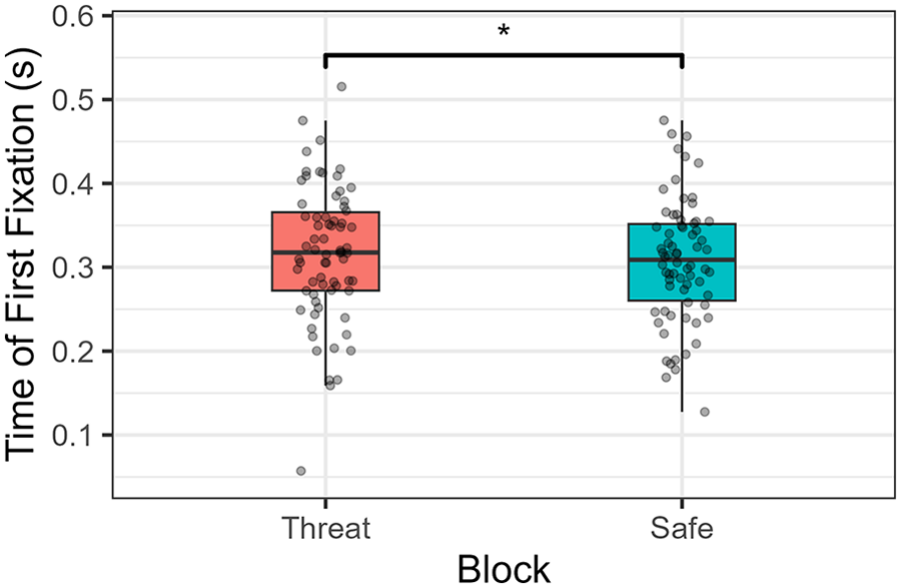
Time until the first fixation after the central action was shown. *p < .05.

When analysing time until first fixation over the central ROI, we can see that, under threat, participants took on average more time to initiate their fixation over this ROI, compared with when they were under safe conditions (χ²(1) = 5.05, *p* = .025; see Figure 9). This longer gaze onset time over the central actions was also observed for neutral expressions compared with fearful faces (χ²(1) = 1,057, *p* < .001). No interaction was observed (χ²(1) = 0.006, *p* = .94).

As for the average fixation number and duration, no effect of block was found in either one of those measurements (χ²(1) = 0.39, *p* = .53, and χ²(1) = 0.5, *p* = .48, respectively).

## Discussion

In this study, we investigated how states of anxiety affect how we perceive potentially aggressive social interactions and to what extent we are able to incorporate external cues to resolve these ambiguous percepts. We used threat of shock to induce anxiety states in participants and gathered decision and timing measures regarding their responses, as well as gaze data.

In terms of decision measures, our analysis showed that, contrary to our Hypothesis (1), threat of shock did not affect sensitivity in identifying aggressive actions. In other words, participants’ ability to correctly identify signal (aggressive actions) among noise was not affected by inducing a state of anxiety. In addition, this was also not moderated by the type of facial expression exhibited by the observer (against our Hypothesis 2). As for criterion, we showed that, in accordance with prior studies (e.g., Manera et al., 2011), when presented with a cue signalling potential aggression (i.e., fearful expression by the observer), participants were more liberal when identifying potentially aggressive actions. Importantly, however, criterion was not affected by threat of shock (against Hypothesis 3), nor was any interaction between this latter factor and the facial expression displayed by the observer.

These results come as a surprise and appear to contradict some previous findings that show a higher sensitivity under threat when detecting both innocuous (de Voogd et al., 2022) and threat-related stimuli (Sussman et al., 2016). The same contradictions are seen for findings regarding criterion, where biases (reduced criterion) towards signalling threat-related stimuli (as opposed to their innocuous counterpart) are reported to be inflated during states of anxiety (Flechsenhar et al., 2022; White et al., 2016). Regardless, it is worth highlighting that some recent studies have shown opposing results in respect to sensitivity (Flechsenhar et al., 2022; Karvay et al., 2022) and criterion (de Voogd et al., 2022). In addition, some studies suggest that the effects of induced threat over sensitivity and criterion are dependent on elevated trait anxiety. This relationship, albeit not observed in our analyses, might be a specific focus of attention in future studies that explore these types of questions (e.g., creating groups of participants with low and high-trait anxiety).

One possible explanation for both the absence of an enhanced sensitivity, as well as a possible reduced criterion towards identifying aggression when under threat, might concern the timeframe of the decision. Most studies measuring sensitivity and criterion parameters do so with paradigms that require/imply fast decision processes. Namely, decisions are made during average time windows that do not usually exceed the 1 to 1.5-s mark (e.g., de Voogd et al., 2022; Sussman et al., 2016). Given the nature of the stimuli used here, that is, complex social interactions, identification times in our study were significantly higher than those studies (around 2.5 s on average and going up to a maximum of around 4.5). One could thus argue that when identification is made to be prolonged (over, for instance, 2 s), and not urged/possible in the first second or so of exposure (like in this study), sensitivity and criterion effects might not manifest themselves to the same extent. In other words, it may be the case that benefits from an enhanced sensitivity and reduced criterion are, usually, mostly valuable when a threat might be imminent. In these cases, a faster ability to detect, even if erroneously, threat-related stimuli, may prove essential in avoiding/minimising unexpected dangerous events (Öhman & Mineka, 2001). This might not be the case, however, when one can more carefully consider/evaluate our surroundings, where more informed decisions might outweigh the benefits of a “quick and dirty” (LeDoux, 1994) visual perception. Thus, although first impressions may capture an enhanced sensitivity and a biased view of threat, a more careful (longer) consideration of the stimulus (such as in this study) might obscure these effects.

Another reason that might help explain the discrepancies found between this and previous studies, concerns how the cues and actions were related. Namely, other studies tend to depict a more direct relationship between cue and target (e.g., “fear” or “F” word followed by a possible fearful expression; Karvay et al., 2022; Sussman et al., 2016). Here, even though participants were instructed that actions and facial expressions were related (observer was presented as reacting to the central interaction), a fearful expression does not necessarily imply/lead to an aggressive interaction as directly as, for instance, the word “aggression” used as prime. As such, in the former studies, one could more clearly establish congruency and incongruency conditions, while in our experiment this relation is not as straightforward.

One final aspect worth noting is that, when assessing the detection of threat-related stimuli, prior studies tend to use facial expressions of either anger or fear, which can more directly imply a threat towards (or relevant to) the observer. Indeed, it has even been shown that fearful and anger expressions are capable of inducing fear-related mechanisms in participants (Springer et al., 2007; Stein et al., 2009; Williams et al., 2005). In our study, we attempted to generalise the effects of anxiety towards other treat-related social stimuli (i.e., aggressive interactions), but which may not implicate a direct threat towards the observer (participant). As such, it can be argued that threat-related sensitivity effects might be directed only towards threat stimuli that more directly signal danger towards the viewer. Future investigations might be useful to explore the difference between these two types of threats.

In what concerns RTs, we showed that being under threat did not contribute to changes in this measure (contradicting Hypothesis 4). Once more, while observed in some studies (Wieser et al., 2010), this finding is not unanimous in the literature, with studies either contradicting (Flechsenhar et al., 2022) or finding no differences between safe and under threat conditions (Robinson et al., 2011). We did see that, on average, aggressive actions were identified more quickly than non-aggressive actions, which aligns with the prior literature showing hastened detection towards threat-related stimuli regardless of anxiety (LoBue & DeLoache, 2010; Öhman & Mineka, 2001).

Drift diffusion model analysis revealed some interesting patterns. The starting point, with neutral facial expressions (compared with fearful expressions) led to starting points closer to the “aggressive” decision threshold. This seems to suggest that participants start their decision making with a greater bias towards signalling aggression when presented with a neutral expression. Furthermore, this effect proved to be moderated by block, with participants under threat expressing a more marked difference in starting points between fearful and neutral facial expressions. This latter effect is surprising but, we believe, can be explained by two factors related to the task itself. First, starting points are inherently hard to capture in this task, since participants were not directly looking at the central ROI when the central action began. Although we computed the RTs as a function of the time of first fixation on this central action, confounding effects regarding the latter (e.g., longer time until first fixation when shown an observer with a neutral expressions) might render these starting point estimates somewhat unreliable. Specifically, by gazing later towards the central action, this might have led DDM estimates to incorrectly assume bias differences between neutral and fearful expressions. This is especially the case since, as shown in our analysis, participants also took longer, on average, to fixate on the central action when under threat. Second, when looking at the full picture that incorporates both starting points and drift rates (Figure 4), it is clear that starting points played little part in the decision process compared with the drift rate (White et al., 2016).

The other effect concerns the increased magnitude of evidence accumulation (drift rate). Specifically, we observed that general accumulation towards the correct response (aggressive or neutral) occurred quicker during the threat of shock condition. This might hint that overall information processing speed and evidence accumulation is improved when under threat, since participants, on average, require less information to reach the correct decision threshold. Thus, it may be a direct reflection of the enhanced sensory–perceptual processing characteristic of anxious states (Cornwell et al., 2017). Indeed, while the literature seems scarce in this topic, studies have shown, for instance, that individuals under threat and with social anxiety have overall higher evidence accumulation rates (Dillon et al., 2022; Gorka et al., 2023). One specific and recent example showed that under threat participants had an overall higher drift rate when perceiving and identifying averted (danger signalling) fearful facial expressions (Beaurenaut et al., 2023). Nevertheless, other studies have instead suggested that anxiety is uniquely associated with an evidence accumulation towards negatively valanced stimuli (Globig et al., 2021; Yamamori & Robinson, 2023), which was not the case here, and should thus be a question for future exploration.

Importantly, since this task was not designed with DDM analysis in mind, the findings discussed above concerning DDM need to be interpreted with care, as they are merely exploratory. This is the case, because, first, DDM analyses were designed for tasks with mean RTs below 1.5 s, which is not the case in this task (*M*_rt_ = 2.53, *SD*_rt_ = 0.65). Nonetheless, recent studies have come to show that the validity of the model parameters extracted from this analysis still hold for tasks with longer RTs (Lerche & Voss, 2019). Finally, and perhaps more importantly, in our task participants did not start the task (to identify aggression) with their visual focus over the target area, a usual requirement from DDM. To overcome this problem, RT was calculated from the time of first fixation over the ROI containing the central action, and not from the time of movie appearance. However, this is still less than ideal, and should be acknowledged.

Gaze analysis showed that under threat, participants had significantly superior dwell times over the observer ROI, particularly in the time windows closest to the appearance of the central actions. That is, participants took longer to gaze away from observer (and towards the central action) under threat of shock compared with safe contexts, which is also in line with the previously discussed difference regarding the time until first fixation over the central action between these blocks. These findings are in line with at least one study showing a later onset for first saccade for emotional faces when under threat of acoustic shock (Flechsenhar et al., 2022). An increased dwell time over faces is also observed in the literature for participants under anxiety and with elevated social anxiety, although this is particularly in cases where the face presents a threat-related expression (fearful or anger expressions; Flechsenhar et al., 2022; Lazarov et al., 2016). It is worth noting, however, that an earlier study conducted by the authors (Silva et al., 2024) found no evidence of superior dwell times over social cues, when these were emotionally neutral. This might suggest that the reason for the increased dwell time during anxious states observed in this study is due to the possible appearance of a threat-signalling cue (fearful expression) in that region. Finally, it is worth noting that no fixation alterations from threat of shock were observed, which, particularly in the case of average fixation duration, goes against findings presented in the same earlier study conducted by the authors. Once more, the emotional nature of the stimuli might be a reason for this discrepancy, or perhaps other factors (e.g., size of the cue’s ROI) might instead be explaining these results. This, however, remains speculative and future studies are needed to clear these findings.

### Limitations

Aside from the limitations already brought forward, some potential concerns should, nonetheless, also be acknowledged. One regards the facial expression exhibited by the observer. While intended to transmit mostly a fearful expression, we ended up with a facial expression that was predominantly recognised as surprise (see the Supplementary Material B). This occurrence (confusing fear for surprise) was, nonetheless, partially expected, as it is backed up by a number of studies (e.g., Kim et al., 2004; Roy-Charland et al., 2014). Since both surprise and fearful reactions are expected facial expressions when viewing a sudden aggressive interaction, we believe that, even if interpreted mostly as surprise, the fearful/surprise facial expressions remain congruent to the aggressive interaction. This is also, at least partially, supported by RT results, showing quicker RTs in identifying aggressive actions when shown a fearful/surprise expression.

Another possible limitation falls upon the attention dedicated towards the facial expressions. Although we controlled the initial attention focus of the participants so that they started each trial by looking at the observer, they might have redirected their attention towards the middle of the screen without actually attending to its facial expression. Eye-tracking data, however, does not seem to support this concern, with participants spending most of their initial time gazing at the face location. Moreover, the data concerning the attention check task showed that participants did, on average, pay attention to the facial expressions exhibited by the observer (see the Supplementary Material C).

Finally, it could be the case that, as presented in the task (under noise techniques), action identification was too difficult, resulting in some random guessing. Although the overall accuracy obtained towards action identification on the main task was around 62%, sitting closely to other similar studies (e.g., Okruszek et al., 2017), and is considerably above random guessing, we cannot assure that the task was properly calibrated to measure, for instance, sensitivity effects. In the same line, the set of aggressive actions used, being depicted in video and through a point-light display, might have failed to evoke the same psychological and physiological activations one would experience in more real-life situations when observing aggressive behaviours between two people. Both these aspects should be considered by future studies.

## Conclusion

We sought to explore if anticipatory anxiety states, induced by threat-of-shock, elicited changes in perceiving aggression on ambiguous social interactions, and how external cues are incorporated during this process. We saw no evidence of an altered perception of these social interactions under anxiety, as well as no change in the ability to use external cues when interpreting them. Nonetheless, anxious states were associated with a faster evidence accumulation towards the correct perceptual decision, suggesting an increased sensory–perceptual processing. Furthermore, an apparent increased gaze dwelling time over external cues was found during the threat condition. Taken together, these findings appear to suggest some limitations to the conclusions brought forward by the previous literature, while also implying other less known effects surrounding anxiety and visual perception. Future research is necessary to better disentangle and understand these incongruencies.

## Supplemental Material

sj-docx-1-qjp-10.1177_17470218241258209 – Supplemental material for The effect of anxiety and its interplay with social cues when perceiving aggressive behaviours Supplemental material, sj-docx-1-qjp-10.1177_17470218241258209 for The effect of anxiety and its interplay with social cues when perceiving aggressive behaviours by Fábio Silva, Marta I. Garrido and Sandra C. Soares in Quarterly Journal of Experimental Psychology
