# Supplementary material for: The effect of anxiety and its interplay with social cues when perceiving aggressive behaviours
Source: Q J Exp Psychol (Hove). 2024 Jul 26;78(6):1124–38. doi: 10.1177/17470218241258209 (PMC12095894; doi:10.1177/17470218241258209)
Supplement: sj-docx-1-qjp-10.1177_17470218241258209 – Supplemental material for The effect of anxiety and its interplay with social cues when perceiving aggressive behaviours [file sj-docx-1-qjp-10.1177_17470218241258209.docx]

Supplementary Material for:

**The effect of anxiety and its interplay with social cues when perceiving aggressive behaviours**

Fábio Silva*^a^, Marta I. Garrido^b, c^, Sandra C. Soares^a^

^a^ William James Center for Research, University of Aveiro, Aveiro, Portugal

^b^ Melbourne School of Psychological Sciences, University of Melbourne, Parkville, VIC, Australia

^c^ Graeme Clark Institute for Biomedical Engineering, The University of Melbourne, Australia

* Corresponding author: Fábio A. Silva, University of Aveiro, Department of Education and Psychology, Universidade de Aveiro, 3810-193 Aveiro.

E-mail: fabio.alexandre@ua.pt

Telephone: 00351 234 370 639

**Supplementary Material A – Bayesian Analyses**

Below we display the full result for the Bayesian analyses conducted for sensitivity, criterion, and response time measures. These analyses were performed in R with the brms package (Bürkner, 2017). We chose non-informative priors for intercepts (Cauchy distribution with x₀ = 0 and γ = 0.2) and betas (Cauchy distribution with x₀ = 0 and γ = 0.5). A total of 2000 iterations (1000 burn-in period) were used in each model. The convergence of the chains was assessed visually (trace plot inspection) and by calculating the Gelman-Rubin statistic. Bayes factors were computed using the bayestestR package for R (Makowski et al., 2019).

*Table 1. Bayesian analysis of sensitivity.*

| Parameter | Est. | Est. Error | l-95% CI | u-95% CI | Rhat | BulkESS | TailESS | BF_01_ |
| --- | --- | --- | --- | --- | --- | --- | --- | --- |
| Intercept | 0.65 | 0.09 | 0.48 | 0.82 | 1 | 3228 | 3236 | <0.001 |
| Block | 0.05 | 0.12 | -0.18 | 0.31 | 1 | 2526 | 2598 | 5.1 |
| Face Emotion | -0.02 | 0.12 | -0.25 | 0.21 | 1 | 2808 | 2830 | 5.21 |
| Block * Face Emotion | 0.03 | 0.17 | -0.31 | 0.35 | 1 | 2366 | 2633 | 3.66 |

*Table 2. Bayesian analysis of criterion.*

| Parameter | Est. | Est. Error | l-95% CI | u-95% CI | Rhat | BulkESS | TailESS | BF_01_ |
| --- | --- | --- | --- | --- | --- | --- | --- | --- |
| Intercept | -0.17 | 0.06 | -0.29 | -0.05 | 1 | 2650 | 2982 | 0.282 |
| Block | -0.03 | 0.08 | -0.20 | 0.48 | 1 | 2478 | 2943 | 6.17 |
| Face Emotion | 0.32 | 0.08 | 0.16 | 0.48 | 1 | 2304 | 3163 | 0.014 |
| Block * Face Emotion | 0.07 | 0.12 | -0.16 | 0.29 | 1 | 2050 | 2812 | 4.69 |

*Table 3. Bayesian analysis of response times.*

| Parameter | Est. | Est. Error | l-95% CI | u-95% CI | Rhat | BulkESS | TailESS | BF_01_ |
| --- | --- | --- | --- | --- | --- | --- | --- | --- |
| Intercept | 2.36 | 0.07 | 2.22 | 2.50 | 1 | 2785 | 3059 | <0.001 |
| Block | -0.03 | 0.10 | -0.22 | 0.16 | 1 | 2434 | 2526 | 7.19 |
| Face Emotion | 0.12 | 0.10 | -0.07 | 0.31 | 1 | 2373 | 2690 | 2.9 |
| Action Emotion | 0.25 | 0.10 | 0.06 | 0.44 | 1 | 2411 | 2731 | 0.167 |
| Block * Face Emotion | 0.03 | 0.13 | -0.21 | 0.28 | 1 | 2233 | 2671 | 4.24 |
| Block * Action Emotion | 0.06 | 0.13 | -0.20 | 0.32 | 1 | 2252 | 2845 | 4.63 |
| Face Emotion * Action Emotion | -0.08 | 0.13 | -0.34 | 0.16 | 1 | 2288 | 2765 | 4.76 |
| Three-way interaction | -0.04 | 0.17 | -0.38 | 0.29 | 1 | 2267 | 2683 | 3.65 |
|  |  |  |  |  |  |  |  |  |

**References**

Bürkner, P.-C. (2017). Brms: An R package for Bayesian multilevel models using Stan. *Journal of Statistical Software, 80*(1), 1–28. <https://doi.org/10.18637/jss.v080.i01>

Makowski, D., Ben-Shachar, M., & Lüdecke, D. (2019). bayestestR: Describing effects and their uncertainty, existence and significance within the Bayesian framework. *Journal of Open Source Software, 4*(40), 1541. https://doi.org/10.21105/joss.01541

**Supplementary Material B – Final Questions**

Below we show some graphical analyses of all three final questions. The first (Figure 10) demonstrates the same emotion identification accuracy for each type of facial expression. Each average (for fearful and neutral) facial expressions were averaged across the two viewing positions/sides (left and right). The second graphic (Figure 11) shows the accuracy distribution regarding the identification (aggressive *vs* non-aggressive) of each action observed during the experimental task. Lastly, the last graph (Figure 12) shows the participant’s judgement on how the relationship between observer (facial expression) and main action was, or was not, dependent on the type of block.


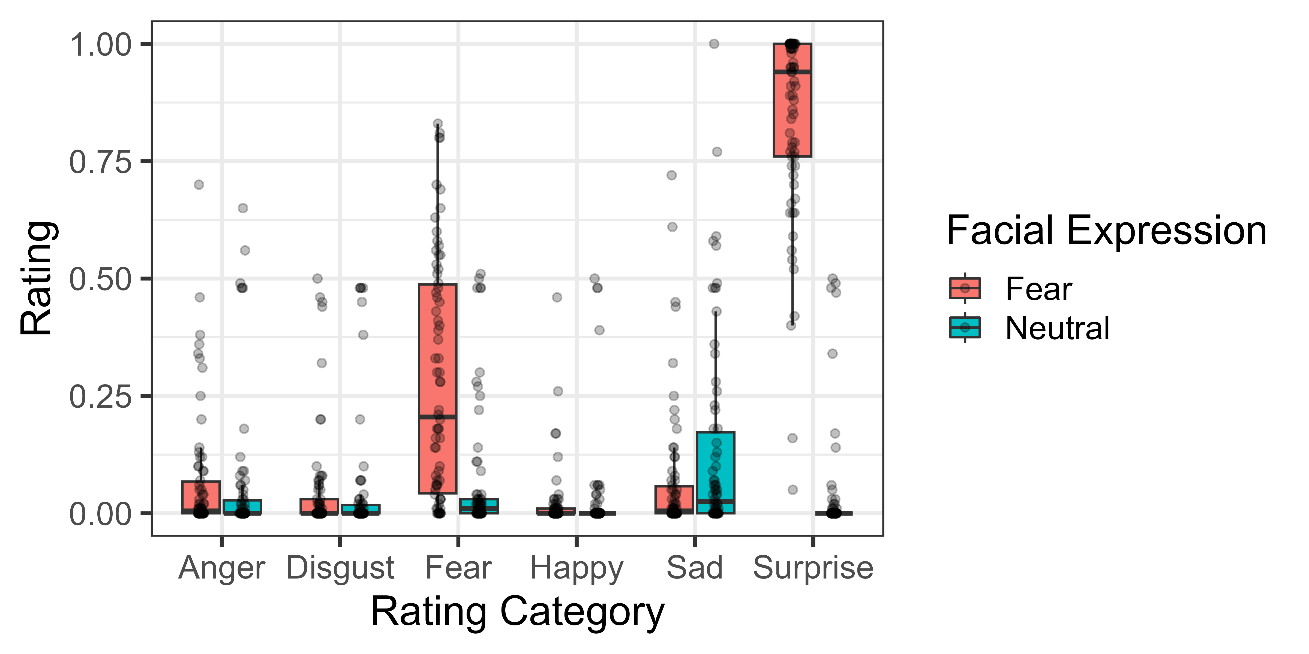


*Figure 10. Distribution of ratings across facial expressions (averaged across left/right variants) across six different possible emotions.*


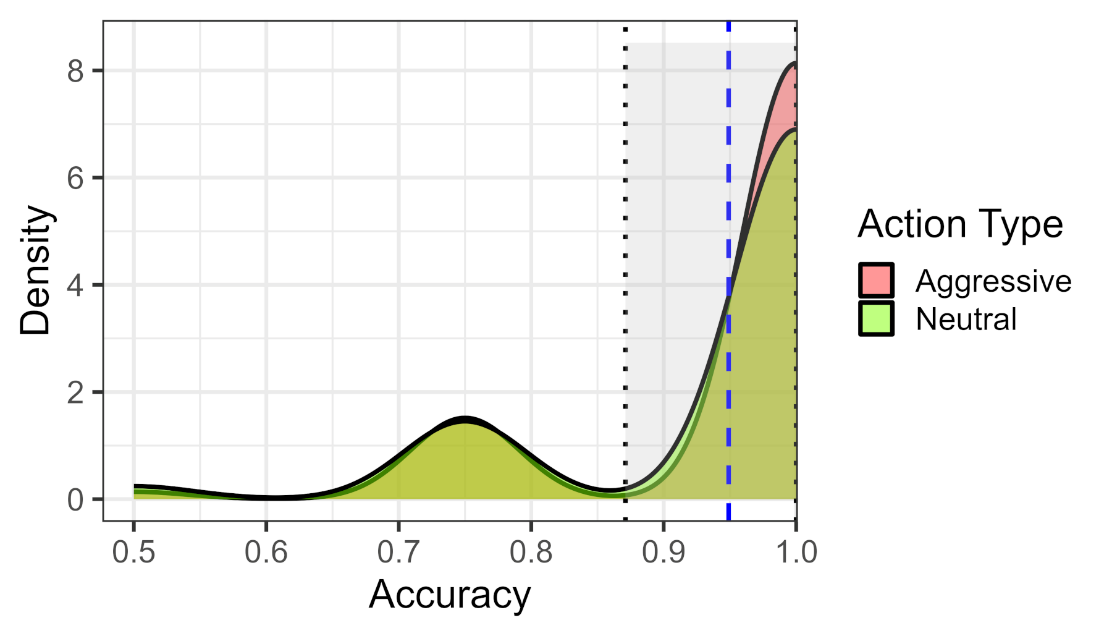


*Figure 11. Emotion identification (aggressive vs neutral) accuracy distribution per Action Type. Blue line represents the overall (regardless of action type) mean. The shadow portion of the figure represents 1 standard deviation range of the mean (trimmed at 1).*


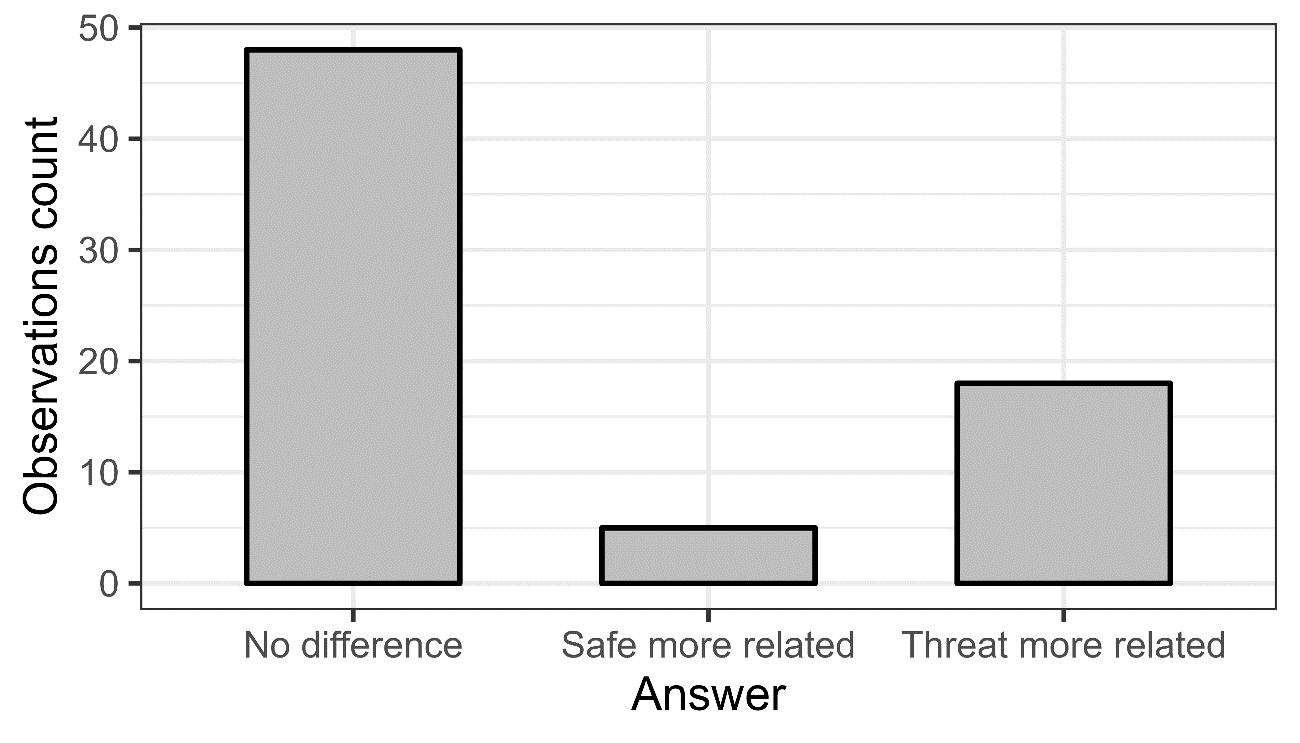


*Figure 12. Count of all answers regarding how the observer and main action were related. Participants could either indicate “no difference” meaning the association between observer and main action was the same across conditions, or “safe more related”/ “threat more related” if the actions were more associated in the safe/threat block.*

**Supplementary Material C – Attention Check**

Below (Figure 13) depicts the distribution of accuracy shown by participants when asked to identify if the observer had performed any sort of facial expression, divided by block. These questions would be prompted at random moments during each block (3 per block, corresponding to around 9% of all trials). Participants had only to select “Z” to indicate that the previous shown observer did exhibit a facial expression (i.e., not a neutral face) or “M” to indicate that no facial expression was made by the observer (no time limit).


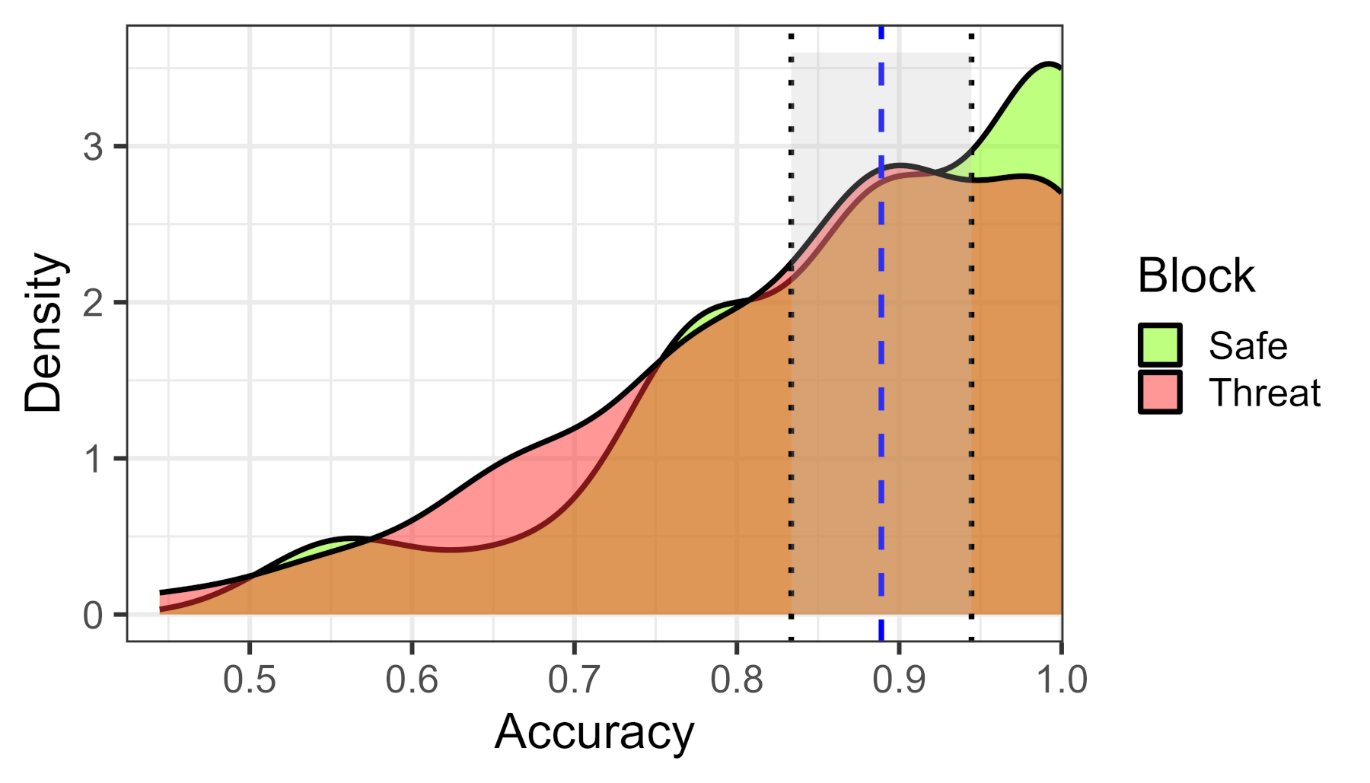


*Figure 13. Accuracy distribution for the attention check task across both safe and threat blocks. The blue line represents the overall (regardless of block) median. The shadow portion of the figure represents the overall interquartile range.*
